# Targeting Endocytosis  and Cell Communications  in the Tumor Immune Microenvironment

**DOI:** 10.1186/s12964-022-00968-3

**Published:** 2022-10-18

**Authors:** Bo Wu, Qian Wang, Xiang Shi, Meixi Jiang

**Affiliations:** 1 Department of General Surgery, The Fourth Affiliated Hospital, China Medical University, Shenyang, 110032 China; 2 Department of Radiology, The Fifth Hospital of Xiamen, Xiamen, 361101 China; 3 Department of Thoracic Surgery, Cancer Hospital of Liaoning Province, China Medical University, Shenyang, 110032 China; 4 Department of Neurology, The Fourth Affiliated Hospital, China Medical University, Shenyang, 110032 China

**Keywords:** Endocytosis, Tumor immune microenvironment, Adhesion molecules, Exosome

## Abstract

The existence of multiple endocytic pathways is well known, and their exact biological effects in tumors have been intensively investigated. Endocytosis can affect the connection between tumor cells and determine the fate of tumor cells. Many relationships between endocytosis and tumor cells have been elucidated, but the mechanism of endocytosis between different types of cells in tumors needs to be explored in greater depth. Endocytic receptors sense the environment and are induced by specific ligands to trigger communication between tumor and immune cells. Crosstalk in the tumor microenvironment can occur through direct contact between cell adhesion molecules or indirectly through exosomes. So a better understanding of the endocytic pathways that control cell adhesion molecules and function is expected to lead to new candidates for cancer treatment. In additional, tumor-derived exosomes may changes immune cell function, which may be a key role for tumors to evade immune detection and response. The overall understanding of exosomes through endocytosis is also expected to bring new candidates for therapeutic regulation of tumor immune microenvironment. In this case, endocytic pathways coordinate cell adhesion molecules and exosomes and can be used as targets in the tumor immune microenvironment for cancer treatment.

**Video Abstract**

**Supplementary Information:**

The online version contains supplementary material available at 10.1186/s12964-022-00968-3.

## Background

Endocytosis refers to the formation of 60–120 nm vesicles through invagination of the plasma membrane, which wraps and imports foreign substances into cells to regulate the internalization of substances (liquid and extracellular components, such as proteins, lipids, metabolites, small molecules and ions), signal transduction and composition [1–3]. The endocytic pathway integrates various signals to promote the development of cells. Receptor-mediated signal transduction can be regulated by endosome sorting, which effectively isolates the receptor from cytoplasmic effectors and promotes proteolysis. Receptor-related processes are more closely related to phosphorylation [4] and ubiquitination levels [5].

**Fig. 1:**
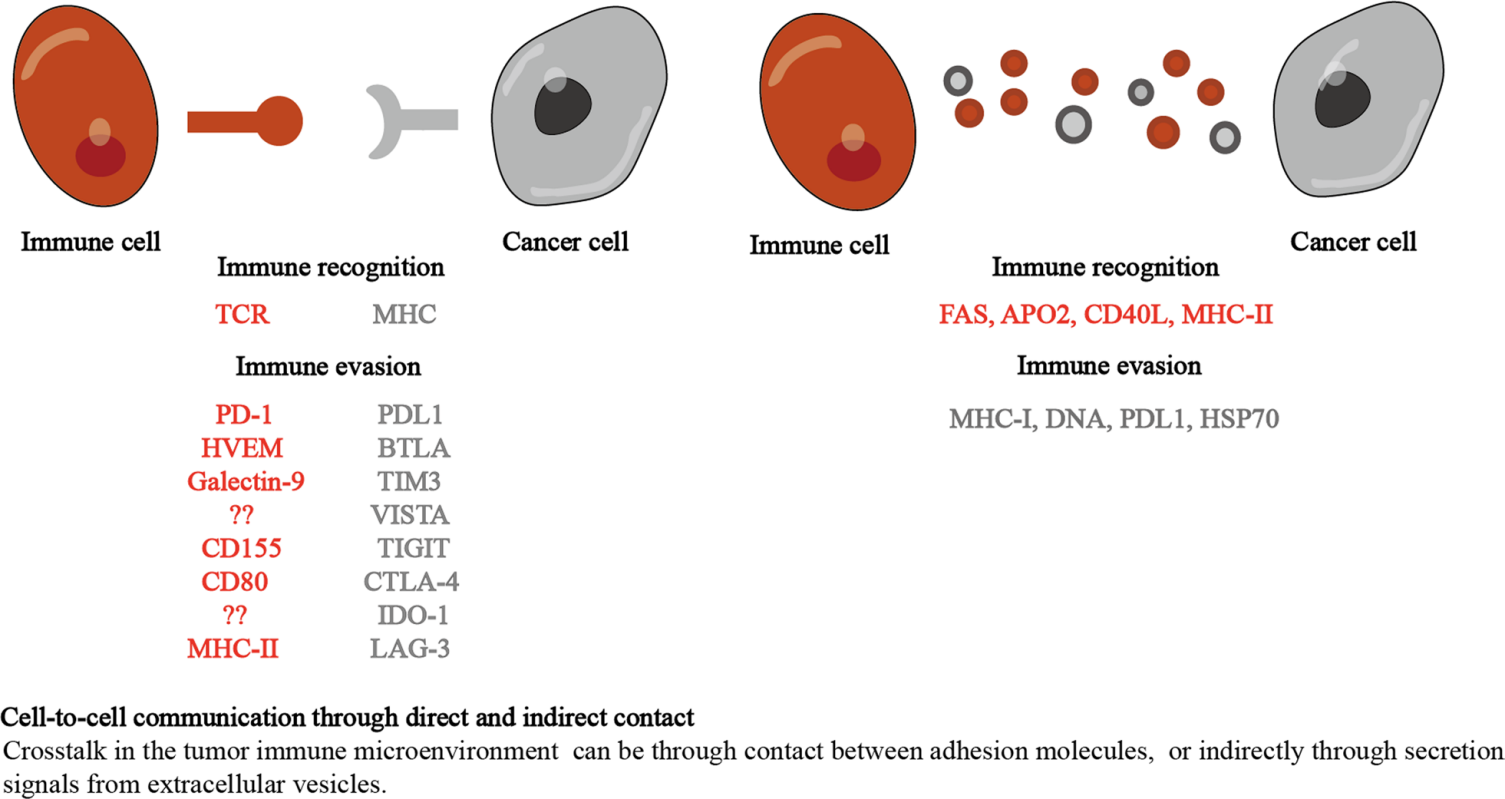
Cell-to-cell communication through direct and indirect contact. Crosstalk in the tumor microenvironment can be through direct contact between adhesion molecules, or indirectly through secretion signals from extracellular vesicles. Tumor surface antigen regulation and tumor-induced immune suppression involve endocytic pathway in tumor immunity. In addition, immunosuppression may arise through accumulating and secreting exosomes around tumor

The well-known effects of endocytosis is necessary for a diverse range of morphogenetic and dynamic tissue events. Endocytosis can cause changes in tissue morphology through various processes, such as signal transduction and effects on the cytoskeleton [6]. Similarly, asymmetric division caused by endocytic transport is an important target for manipulating stem cells that lead to tumor recurrence [7]. In addition, endocytosis and different types of cells intertwine to play a decisive role in the tumor microenvironment (TME). The crosstalk in the tumor microenvironment can occur directly through cell-to-cell contact between cell adhesion molecules or indirectly through extracellular vesicles. Immune cells, including specialized antigen-presenting cells and natural killer cells, rely on endocytosis to quickly gather receptors to detect targets on tumor cells for antigen presentation [8]. Exosomes target specific types of recipient cells, and the exchange of information between cells also involves endocytosis [9]. Therefore, endocytosis mediates the communication between tumor cells and immune cells and coordinates the interaction between different types of cells to control the tumor immune microenvironment (Fig. 1). We review and clarify the role of endocytosis in tumor cells and the latest developments in communication in the tumor microenvironment.

## Progression of endocytosis in tumor cells

Studies in the past decade have shown that the functional interaction between cell signaling and endocytosis is important at all stages of morphogenesis and regulating cell proliferation, metabolism, movement, differentiation and immunity [10]. Cells sense the environment and each other through activation of cell surface signal receptors induced by ligands. Among them, tyrosine kinase receptor (RTK) and G protein-coupled receptor (GPCR) participate in homeostatic regulation to prevent ligand-induced overactivation of downstream effectors. This paradigm has also been extended to other receptors, including transforming growth factor (TGFβ) and cytokines. In addition, Notch and Wnt coordinate the fate of adjacent tumor cells through endocytosis, highlighting the influence of cell morphology on fate [11, 12]. Endocytosis seems to be the simplest way to regulate cell signal transduction by controlling the number of activated receptors. The activation of receptors or downstream effectors usually stimulates endocytosis, but questions remain about endocytosis and signal transduction under in vivo conditions. Endocytosis and signal transduction seem to be two aspects of the same coin, raising the question of whether the same biochemical pathway can achieve different biological results. Similarly, given that the high overlap between pathways is activated by multiple signal receptors, can the detection mechanism on the cell membrane break down many input signals into specific signals?.

The increasing understanding of the link between endocytosis and signal transduction raises the possibility that targeted interference with endocytosis may alter disease-related phenotypes, especially those related to abnormal cell specifications. The endocytosis mechanism in tumor heterogeneity may be the basis of the specific characteristics of tumors and their level of sensitivity to therapeutic drugs targeting signal receptors [13]. The dynamic balance in tissues strongly depends on the interaction between cells and the extracellular matrix [14]. In contrast, integral proteins can regulated the extracellular matrix (ECM) and transmit signals between the cell and its surroundings [15].

In the past decades of research, the main focus has been on studies related to endocytosis and signaling pathways. With a better understanding of the tumor immune microenvironment, the relationship between tumor cells and immune cells is now recognized, and endocytosis mediates cell-to-cell communication through the regulation of direct or indirectly contact. Therefore, we discuss in depth about endocytosis mediating tumor immune microenvironment through regulation of cell adhesion molecules (including major histocompatibility complex (MHC), immune checkpoints) and exosomes.

## Endocytosis mediates tumor immune microenvironment through cell adhesion molecules

### Endocytosis and tumor immune microenvironment

The overall complexity of tumors presents challenges to the development of effective anticancer treatments [16–18]. In the process of tumor development, tumor heterogeneity intensifies as tumor cells and noncellular components of the tumor microenvironment (TME) mature [19, 20]. The TME consists of extracellular matrix (ECM), stromal cells (such as fibroblasts, mesenchymal stromal cells, pericytes, occasionally fat cells, blood and lymphatic network) and immune cells (including T and B lymphocytes, natural killer cells, macrophages) [21]. Tumor immune escape refers to the ability of tumor cells to avoid recognition and attack by the immune system. It is an important strategy for tumor survival and development [22]. Tumor surface antigen regulation and tumor-induced immune suppression involve the endocytic pathway in tumor immunity.

The cells of the innate immune system, such as monocytes, macrophages and dendritic cells (DCs)—are specialized antigen-presenting cells. In addition to this natural killer cells (NKs) rely on recognition of receptors and other cell surface molecules to rapidly detect microbial proteins or membrane molecules on tumor cells to orchestrate downstream inflammatory responses [23]. Key to the bridging role between innate and adaptive immunity is the processing and cross-presentation of antigens by APCs to T cells. The ability of APCs to engulf tumor cells through phagocytosis, a process that involves target cell recognition, phagocytosis and lysosomal digestion, is regulated by receptor-ligand interactions. Although healthy normal tissues and cells inherit the ability to avoid self-clearance by phagocytosis by expressing anti-phagocytic molecules, cells are more dependent on similar mechanisms to evade immune eradication [24, 25]. Thus identifying and targeting phagocytic checkpoints in cancer will provide a new avenue to develop cancer immunotherapies to eliminate tumor immune escape.

More and more phagocytic checkpoints are found to play an essential role in innate and adaptive immunity. Phagocytic checkpoint blockade, including anti-CD47 therapy and PD-L1 blockade, stimulates the innate and adaptive immune systems to generate anti-tumor responses, combining them with existing cancer immunotherapy strategies to improve the response rate to tumor treatment [26]. When major signaling pathways are constitutively activated by genetic disorders, such as v-Src or mutated K-Ras, a receptor-independent pattern of macropinocytosis occurs. Macropinocytosis provides tumor cells with an additional means of acquiring nutrients and internalizing adhesions molecules to support their growth and spread. By inhaling and concentrating amino acids and proteins in the extracellular fluid, tumor cells activate the mammalian target of rapamycin 1 (mTORC1) to stimulate transcriptional translation and support growth [27]. Thus, endocytosis inhibitors as well as immune checkpoint blockade therapy offer promise for clinical trials in a wide range of tumors, and can be used in combination with other monoclonal antibodies or immune checkpoint inhibitors (Table 1).

**Table 1:** Categorization and features of endocytosis process

Endocytosis process	Associated protein	Mechanical	Inhibitor	Endocytosis checkpoint
Clathrin-mediated endocytosis	Actin	Membrane tension	Clorpromazine	E-, N-, and VE-cadherin, integrins, Notch, RTKs (EGFR, Her2, and FGFR1), Wnt, GPCR
	Clathrin	Membrane tension		
	ENTH domain	Membrane tension		
	N-BAR	Membrane tension		
Caveolae-mediated endocytosis	Cav-1	Low shear stress	Methyl-cyclodextrin	
	Cavin-1	Membrane stretch		
	Filamin A	Loss of cell adhesion		
Clathrin/caveolae-independent endocytosis	GPI-anchored	Membrane tension		Integrins, Notch, RTKs(EGFR, Her2, and FGFR1), Wnt, GPCR
	Vinculin	Membrane tension		
	TORC2	Membrane tension		
Macropinocytosis	Rac1 and CDC42	Aspect ratio of cargo	EPIA, amiloride	MHCI, MHC-II, mTORC1
	Phosphatidic acid	Membrane stretching		
	PLD2	Membrane tension		
	SCAR/WAVE	Actin-nucleation-promoting factors		
	WASp/N-WASp	Actin-nucleation-promoting factors		
Phagocytosis	Rac1	Substrate stiffness		CD47-Signal-regulatory protein α (SIRPα), PDL1, MHC I-LILRB1
	Cdc42	Substrate stiffness		
	MRTF-A	Area confinement		
	TRPV4	Substrate stiffness		

### Endocytosis and cell adhesion molecules

The differentiation of initial T cells into effector cells can promote the killing of cancer cells. This effect occurs when the T-cell receptor (TCR) triggered by the signal accumulates, and then specific antigen presenting cells (APCs) are recognized [28]. The imbalance of endocytic events that control TCR circulation and degradation has been considered an important determinant of antigen presentation by immune cells. TCR is a protein complex formed by an antigen recognition module composed of α and β chains and a signal transduction module composed of ζ chain homodimers and CD3 chain clusters [29].

**Fig. 2:**
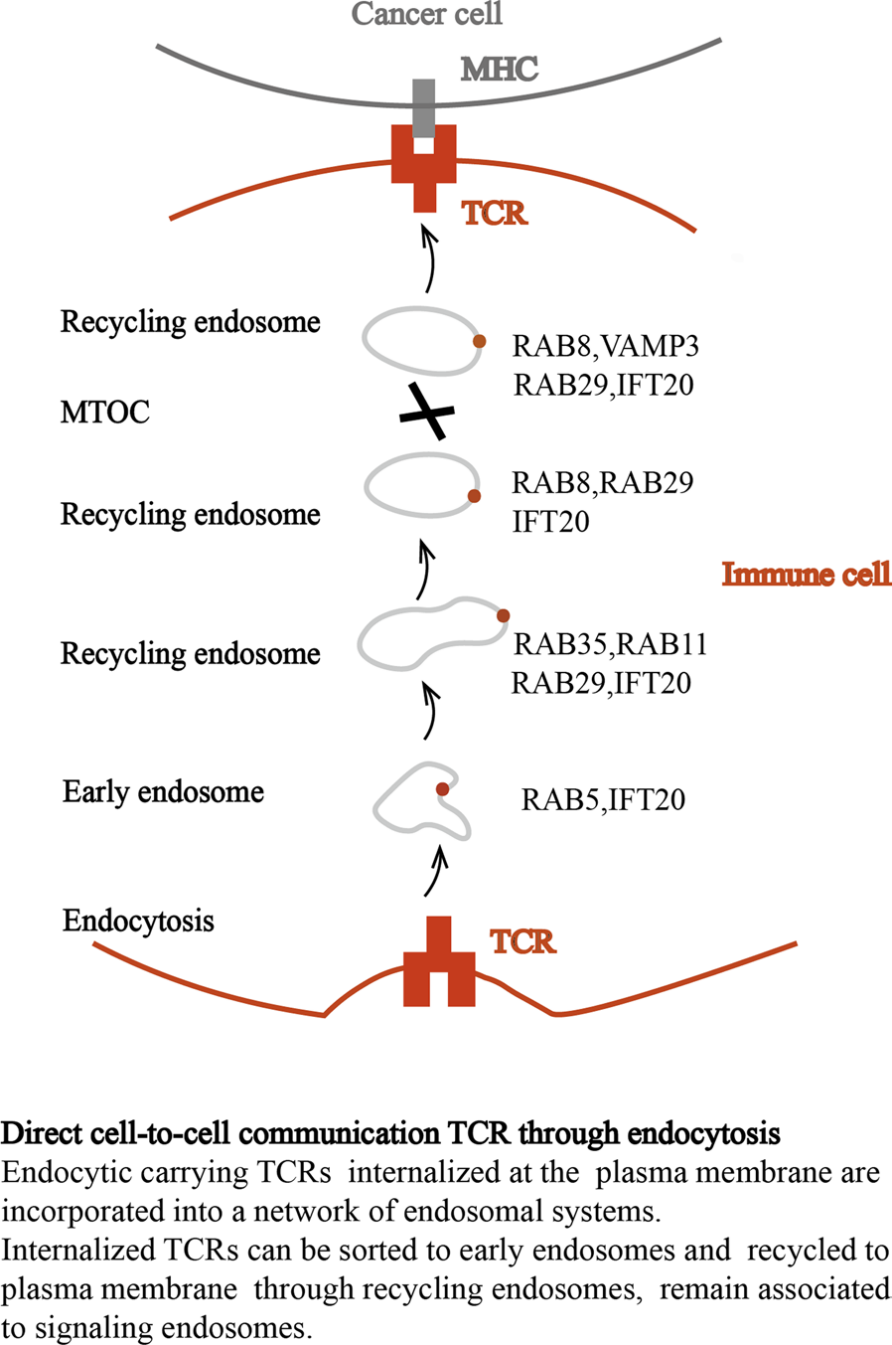
Regulation of immune response by exosomes. Exosomes from distinct cellular sources, including immune cells (B cell, T cell, macrophage, and dendritic cells) and cancer cells, exosomes with cargos that can influence the proliferation and activity of recipient cells of both the innate and adaptive immune system

At present, clathrin-dependent and clathrin-independent endocytosis have been identified as the main pathways involved in the internalization of TCR [30]. Postendocytosis receptor movement is coordinated by ubiquitinated Rab GTPases, SNARE and regulators and effectors of endosomal subpopulations [31, 32]. The cargo can be recovered directly from early endosomes (ESEs) via a rapid, microtubule-independent process is achieved by rabenosyn5, which is the Fab 1, YOTB, Vac 1 and EEA1 (FYVE) domain containing Rab5 and Rab4 effectors [33]. Internalized receptors are incorporated into endosomes and can also be delivered to the plasma membrane through a slow, microtubule-dependent pathway [34]. In addition to the universal Rabs, Rab3d, Rab8a, Rab8b, Rab29, Rab35, intraflagellar transport (IFT), and electrohydrodynamic (EHD) family proteins act sequentially in this pathway based on the ability to recycle TCRs [35–37]. Rab8 has been identified as the terminal pathway. It recruits v-SNARE VAMP3 and t-SNARE SNAP23 synaptic fusion protein to finally allow the recovered TCR to be fused to the cell membrane [36]. T cells can also enhance the release of extracellular vesicles (EVs) through stimulation, such as TCR triggering or T-cell activation [38, 39]. Activated T cells release biologically active Fas ligand and APO2 ligand in EVs, thereby promoting activation and inducing cell death [40]. In addition, the EVs formed by CD8^+^ CTL MVBs contain granzyme and perforin [41] (Fig. 2).

To achieve complete activation, B cells rely on their ability to capture external antigens and present them to CD4^+^ T cells as peptide fragments loaded on major histocompatibility complex class II (MHC II) molecules [42]. This interaction differentiates B cells into plasma cells that produce high affinity and develop into memory B-cell populations [43]. Regarding the mechanism by which B cells extract antigens on the cell surface, one view is that local lysosomes secrete and release proteases and acidify the synaptic cleft of related antigens to facilitate their extraction of antigen [44]. Another view is that the tension exerted on the synaptic membrane mediated by myosin II-A triggers internalization of the antigen into coated clathrin [45].

The binding of surface antigens to the B-cell receptor (BCR) triggers the recruitment of PAR3 to the antigen contact site, which leads to polarization of the microtubule network, in which the centrosome transfers to the immune synapse in a Cdc42-dependent manner [44, 46]. Centrosome relocation directs the recruitment of MHC II^+^ lysosomes, which can fuse with antigen-containing endosomes to facilitate antigen processing. It is worth noting that the Lamp1^+^ multivesicular compartment, which contains both antigen and MHC molecules, has been found to be closely related to the immune synapse of activated B lymphocytes [44]. Therefore, determining the specific mechanism used to selectively enhance the extraction of antigens by B cells to enhance the activation of T cells should be the focus of future research.

Immunosuppression involves inducing the expression of immunosuppressive molecules or their receptors, including cytotoxic T-lymphocyte-associated protein 4 (CTLA-4), programmed cell death protein 1 (PD-1), T cell immunoglobulin and mucin domain 3 (TIM-3), Indoleamine 2,3-dioxygenase (IDO), V-domain Ig inhibitor of T-cell activation (VISTA), killer cell immunoglobulin-like receptors (KIR), T cell immunoglobulin and ITIM domain (TIGIT), B and T lymphocyte attenuator (BTLA) and Lymphocyte activation gene-3 (LAG-3), which are called immune checkpoints and can inhibit the activated lymphocytes of effector T cells and ultimately lead to tumor immune escape [47]. Immune checkpoints are also specifically expressed on protumor immune cells (e.g., Tregs). For example, PD-1 on T effectors reduces activation, while PD-1 on Tregs enhances immunosuppressive effects. In addition, linker for activation of T cells (LAT) [48, 49] and lymphocyte-specific protein tyrosine kinase (LCK) [50, 51], with the assistance of specific vesicle-related proteins, ensure the optimal TCR level required for T-cell activation. Changes in endocytic transport are associated with cancer, so a better understanding of the endocytic pathways that control immune checkpoints and function is expected to lead to new candidates for cancer treatment.

## Endocytosis mediates tumor immune microenvironment through exosomes

### Endocytosis and exosomes

In addition, immunosuppression may arise through the accumulation and secretion of exosomes around tumors. Exosomes can inactivate cytotoxic T lymphocytes (CTLs) to enhance the immune tolerance of tumor cells [52–55]. The communication between cancer cells and surrounding cells is a bidirectional process that involves multiple mechanisms. Crosstalk in the tumor microenvironment can occur directly through contact between antigen presentation or indirectly through secretion signals from extracellular vesicles. Therefore, the therapeutic method of regulating cell-to-cell communication by endocytosis may be a promising strategy in the fight against tumors.

**Fig. 3:**
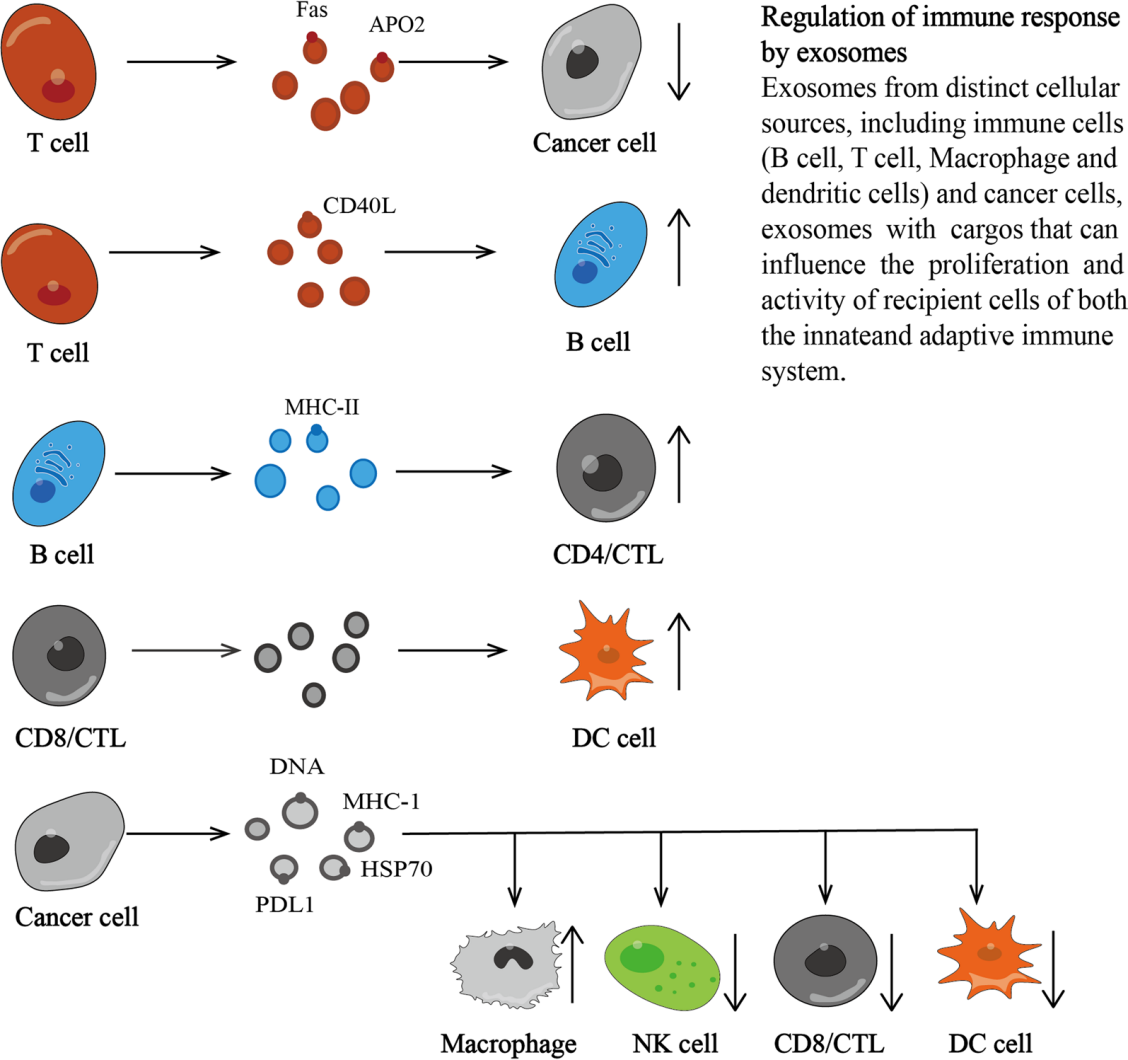
Regulation of immune response by exosomes. Exosomes from distinct cellular souurces, including immune cells (B cell, T cell, Macrophage and Dendritic cells) and cancer cells, exosomes with cargos than can influence the proliferation and activity of recipient cells of both the innateand adaptive immune system

Liquid and extracellular components (such as proteins, lipids, metabolites, small molecules and ions) can enter cells through endocytosis and plasma membrane invagination, along with cell surface proteins [56]. Tumor-derived exosomes are bound and internalized by organ-specific cells. Heparan sulfate proteoglycans mediate the interaction between cells and exosomes. Exosome transfer to the recipient cell can be competitively blocked by heparinoids because heparin is structurally similar to heparan sulfate [57]. The plasma membrane bud formed on the side of the cell cavity has an orientation from outside to inside, which leads to the formation of the ESE (early endosome) [58]. The ESE can also be fused with the ER (endoplasmic reticulum) and anti-Golgi network (TGN), which may explain why the phagocytic cargo contains components of the ER, TGN and mitochondria, and the ESE may contain membrane and intraluminal components representing different origins [58]. MVBs are formed by the inward invagination of the late endosome restriction membrane (that is, the two invaginations of the plasma membrane). MVBs contain multiple intraluminal vesicles (ILVs), which lead to exosomal cargo in future modifications. As part of the formation of ILVs, proteins (originally located on the cell surface) can be clearly distributed between ILVs [56]. MVBs can be fused with autophagosomes, and the final content will be degraded in the lysosome, allowing the degradation products to be recovered by the cell. MVB that does not follow this trajectory is transported to the plasma membrane through the cell cytoskeleton and microtubule network and is docked on the lumen side of the plasma membrane with the help of MVB docking protein to cause exocytosis [59]. Rabs, endosomal sorting complex required for transport (ESCRT) and other related proteins (CD9, CD81, CD63, TSG101, Alix, and putative universal biomarker of syntenin-1) are used as exosomal markers or are related to the biogenesis of exosomes [58, 60] (Fig. 3).

Exosomes can also contain different types of cell surface proteins, intracellular proteins, RNA, DNA, amino acids and metabolites [56]. The questions surrounding the function of exosomes focus mainly on understanding the fate of their components and their induction of phenotypic and molecular changes in recipient cells. The uptake and secretion pathways of exosomes may intersect, resulting in a mixed population of endogenously produced and circulating exosomes produced over time. The unique mechanisms and pathways related to the uptake of exosomes [58, 61], as well as the specificity of exosomes for certain cell types, increase the functional complexity of exosomes in cell-to-cell communication.

Exosomes are vesicles with membrane structures between 40 and 160 nm (both 100 nm) in diameter [9, 62, 63] containing RNA, proteins and lipids that play a role in tumor proliferation, metastasis, immunosuppression and drug tolerance [58, 61]. These processes seem to be similar to leukocyte transendothelial migration, in which integrins are involved in the adhesion/attachment of exosomes to receptor cells, followed by the enrichment of four transmembrane microstructural domains facilitating exosome fusion [64–67]. The endocytosis of exosomes is the most important way they deliver content. It can be divided into micropinocytosis [68, 69], phagocytosis [70], clathrin-mediated endocytosis [71], caveolin-mediated endocytosis [72] and clathrin/caveolin-independent endocytosis [73]. The endocytosis of exosomes depends on the actin cytoskeleton, phosphatidylinositol 3-kinase (PI3K) and dynamin2 [70]. Studies have shown that the pharmacological inhibitors EIPA and LY294002 inhibit Na^+^-H^+^ ion exchange and PI3K activity, which can inhibit the effect of macropinocytosis and reduce the uptake of exosomes [74]. Clathrin-dependent endocytosis uses clathrin and AP2 to cover the membrane and induce exosomes to invade vesicles; clathrin/cavolin-independent endocytosis is caused by RhoA, Cdc42 and Arf6 [75].

Exosomes targeting recipient cells by endocytosis have been confirmed in tumors. For example, oncogenic signals induced by KRAS mutation expression promote exosomal uptake in human pancreatic cancer cells through micropinocytosis [2, 76] and promote the uptake of exosomal cargo by human melanoma cells by fusion with the plasma membrane [77]. Exosomes derived from rat adrenal medulloma PC12 cells are more likely to rely on clathrin-dependent endocytic uptake [74]. It is possible that internalized exosomal cargo varies depending on the endocytosis and the recipient cell status that regulates uptake of extracellular molecules and vesicles.

### Exosomes in tumor progression and metastasis

The discovery of exosomes, especially their role in mediating the transportation or “trafficking” of biological materials, has explained various pathological and physiological phenomena that involve the transmission of information between cells [78]. As a new model for mediating information exchange between cells, exosomes transport oncogene message during the occurrence and development of tumors. Recent studies have elaborated on the important role of exosomes in tumor carcinogenesis [76]. Tumor-derived exosomes can promote tumor formation by regulating the synthesis of cell-independent ncRNA [79]. During the development of cancer, there is competition between cancer cells and neighboring normal cells [80]. As a homeostatic mechanism, abundant noncancer cells can release tumor suppressor miRNAs, thereby suppressing the malignant phenotype of adjacent cancer cells [81–86]. In addition, it has been reported that differences in exosome content can distinguish several types of cancer cells (such as prostate cancer, gastric cancer, and laryngeal squamous cell carcinoma) from normal cells [87].

Exosomal RNA derived from tumor cells can enhance the proliferation, migration and tube formation of endothelial cells, thereby promoting tumors and lymphatic vasculature [88–93]. Proteomic analysis of exosomes showed that the integrin expression pattern of cancer cells contributes to the tendency of metastasis [94]. For example, integrin α6β4 and α6β1 are related to lung metastasis, and integrin αvβ5 is related to liver metastasis [95]. Depletion of integrins α6β4 and αvβ5 reduced exosomal uptake and resulted in the inhibition of lung and liver metastasis, respectively. Therefore, the integrins found on specific tumor-derived exosomes can be used to predict organ-specific cancer metastasis and are a new target for the development of cancer metastasis treatment strategies [96–99].

### Exosomes regulate cancer immunology

In most studies, the recipient cells of tumor derived exosomes are cancer-related immune cells and other stromal cells, which dynamically regulate each other in the tumor microenvironment [100]. Compared with studying the role of exosomes in other types of cells, research on tumor related exosomes is progressing rapidly. More and more evidence supports the complex intercellular communication mediated by exosomes in tumor immune microenvironment. Tumor-derived exosomes content HSP72 can trigger myeloid-derived inhibitory cell activation through STAT3 [101]. Tumor exosomes block the maturation and migration of dendritic cells in a PD-L1 dependent manner [102]. The tumor-derived exosomal DNA by circulating neutrophils can enhance the production of tissue factor and IL-8, thereby promoting tumor inflammation and thrombosis [103]. Therefore, tumor-derived exosomes may changes immune cell function, which may be a key role for tumors to evade immune detection and response.

Similarly, exosomes released by immune cells affect tumor development by regulating immune response [104]. Exosomes released by NK cells show FasL membrane expression, and produce strong cytotoxicity to cancer by eliminating Fas + tumor cells [105]. In addition, in patients with acute myeloid leukemia (AML), plasma exosomes carrying leukemia-related antigens and a variety of inhibitory molecules can inhibit tumor activity by interfering with NK-92 cells [106]. NK-92 cell-derived exosomes TNF-α have cytotoxic effects on melanoma cells and block cell proliferation signaling pathways [107]. In a phase II trial, IFN-γ mature DC-derived exosomes loaded with MHC class peptides can enhance NK cell activity in patients with non-small cell lung cancer (NSCLC) [108]. T cells can also transfer CD40L to B cells through helper T cells [109]. The binding of antigen-loaded B cells to specific CD4^+^ T cells stimulates the release of EVs with peptide MHC-II complexes, which directly stimulate naive CD4^+^ T cells [110] (Fig. 3). In addition, ovalbumin (OVA)-stimulated dendritic cell exosomes are more effective than microvesicles to trigger antigen (OVA)-specific CD8^+^ T cell activation [111].

## Conclusion

The field of communication in the tumor microenvironment is a relatively new concept in tumor biology and rapidly evolving. Cancer-stromal crosstalk is an extremely complex phenomenon, and different forms of cellular communication are highly expressed in cancer and clearly involved in cancer development. Different forms of cell communication are highly expressed in cancer and obviously participate in the occurrence of cancer. With our in-depth exploration and understanding of the connection of endocytosis, we believe that the communication between cells is essential for the creation of tumor niches. Therefore, a novel medical method focuses on inhibiting cell-to-cell communication in cancer, or using these communication methods as a vehicle for delivering drugs to tumor cells. Immune cells can rely on endocytosis to mediates cell adhesion molecules quickly detect targets on tumor cells. The overall understanding of exosomes through endocytosis is also expected to bring new candidates for therapeutic regulation of tumor immune microenvironment. Therefore, further research is needed to fully understand endocytosis and clarify possible specific targets to inhibit tumors.

## Data Availability

Not applicable.

## Abbreviations

TME: Tumor microenvironment
ILVs: Intraluminal vesicles
RTK: Tyrosine kinase receptor
GPCR: G protein-coupled receptor
TGFβ: Transforming growth factor
ECM: Extracellular matrix
TCR: T cell receptor
BCR: B cell receptor
MHC: Major histocompatibility complex
CTLA-4: Cytotoxic T-lymphocyte-associated protein 4
PD-1: Programmed cell death protein 1
TIM-3: T cell immunoglobulin and mucin domain 3
IDO: Indoleamine 2,3-dioxygenase
VISTA: V-domain Ig inhibitor of T-cell activation
KIR: Killer cell immunoglobulin-like receptors
TIGIT: T cell immunoglobulin and ITIM domain
BTLA: B and T lymphocyte attenuator
LAG-3: Lymphocyte activation gene-3
CTL: Cytotoxic T lymphocytes
Treg cells: Regulatory T cells
MDSC: Marrow-derived suppressor cells
DC: Dendritic cells
NK: Natural killer
mTORC1: Mammalian target of rapamycin 1
APC: Antigen presenting cells
ESE: Early endosome
FYVE: Fab 1, YOTB, Vac 1 and EEA1
IFT: Intraflagellar transport
EHD: Electrohydrodynamic
LCK: Lymphocyte-specific protein tyrosine kinase
LAT: Linker for activation of T cells
EVs: Extracellular vesicles
MVBs: Multivesicular bodies
ER: Endoplasmic reticulum
TGN: Anti-Golgi network
ESCRT: Endosomal sorting complex required for transport
PI3K: Phosphatidylinositol 3-kinase
PS: Phosphatidylserine
FasL: Fas ligand
AML: Acute myeloid leukemia
NSCLC: Non-small cell lung cancer
OVA: Ovalbumin
SC: Stem cell
IC: Immune checkpoint
MSI-H: Microsatellite instability
dmmR: DNA mismatch repair defect
HLA-B: Human leukocyte antigen B
CEACAM-1: Carcinoembryonic antigen cell adhesion molecule 1
PVR: Poliovirus receptor
